# Copy number analysis of *NIPBL* in a cohort of 510 patients reveals rare copy number variants and a mosaic deletion

**DOI:** 10.1002/mgg3.48

**Published:** 2013-11-14

**Authors:** Yu-Wei Cheng, Christopher A Tan, Agata Minor, Kelly Arndt, Latrice Wysinger, Dorothy K Grange, Beth A Kozel, Nathaniel H Robin, Darrel Waggoner, Carrie Fitzpatrick, Soma Das, Daniela del Gaudio

**Affiliations:** 1 Department of Human Genetics, University of ChicagoChicago, Illinois; 2 Department of Pathology, University of ChicagoChicago, Illinois; 3 Department of Pediatrics, Division of Genetics and Genomic Medicine, Washington University School of MedicineSt. Louis, Missouri; 4 Department of Genetics, University of Alabama at BirminghamBirmingham, Alabama

**Keywords:** Array-CGH, copy number variation, Cornelia de Lange, MLPA, *NIPBL*

## Abstract

Cornelia de Lange syndrome (CdLS) is a genetically heterogeneous disorder characterized by growth retardation, intellectual disability, upper limb abnormalities, hirsutism, and characteristic facial features. In this study we explored the occurrence of intragenic *NIPBL* copy number variations (CNVs) in a cohort of 510 *NIPBL* sequence-negative patients with suspected CdLS. Copy number analysis was performed by custom exon-targeted oligonucleotide array-comparative genomic hybridization and/or MLPA. Whole-genome SNP array was used to further characterize rearrangements extending beyond the *NIPBL* gene. We identified *NIPBL* CNVs in 13 patients (2.5%) including one intragenic duplication and a deletion in mosaic state. Breakpoint sequences in two patients provided further evidence of a microhomology-mediated replicative mechanism as a potential predominant contributor to CNVs in *NIPBL*. Patients for whom clinical information was available share classical CdLS features including craniofacial and limb defects. Our experience in studying the frequency of *NIBPL* CNVs in the largest series of patients to date widens the mutational spectrum of *NIPBL* and emphasizes the clinical utility of performing *NIPBL* deletion/duplication analysis in patients with CdLS.

## Introduction

Cornelia de Lange syndrome (CdLS [MIM 122470]) is a multisystem disorder characterized by characteristic facial features, growth retardation, intellectual disability, limb reduction defects, hirsutism, and moderate-to-severe neurodevelopmental delay (Kline et al. 1993). There is marked heterogeneity in the CdLS phenotype. At one end of the spectrum are individuals with classical CdLS features exhibiting profound growth and neurodevelopmental delay, sometimes accompanied by severe limb defects. Less severe growth retardation and developmental delay have been observed in mildly affected individuals. The prevalence of CdLS is estimated to be 1:10,000 live births, but the incidence may be underestimated given the existence of undiagnosed individuals with milder phenotypes.

Mutations in the *NIPBL* gene (MIM 608667) have been identified in ∼60% of classical CdLS patients (Krantz et al. 2004; Tonkin et al. 2004). *NIPBL*, located on chromosome 5p13, encodes for the human ortholog of *Drosophila Nipped-B* belonging to the family of chromosomal adherins, which are regulators of chromatin cohesion and enhancer–promoter communication in *Drosophila* (Rollins et al. 1999, 2004). Genotype–phenotype correlation studies have demonstrated that *NIPBL* mutation-positive patients tend to have a more severe phenotype than mutation-negative patients with truncating mutations, generally causing a more severe phenotype than missense mutations based on limb differences, growth, and cognitive function (Gillis et al. 2004). This suggests that *NIPBL* is a dosage-sensitive gene. Recently, somatic mosaicism of *NIPBL* mutations has been described as a relatively frequent occurrence (Huisman et al. 2013). Mutations in *SMC1A* (MIM 300040) and *SMC3* (MIM 606062) account for ∼5% of patients with a milder variant of CdLS (Musio et al. 2006; Deardorff et al. 2007). More recently, de novo mutations in *RAD21* (MIM 614701) and *HDAC8* (MIM 300269) have been identified in individuals with growth retardation, minor skeletal anomalies, and cognitive and facial features consistent with those caused by mutations in *NIPBL* (Deardorff et al. 2012a,b2012b). The molecular etiology of the remaining CdLS cases remains unknown.

While the majority of mutations in Mendelian disorders are detected by sequence analysis, intragenic deletions and duplications are becoming an increasingly significant factor in elucidating the molecular etiology of many of these conditions (Aradhya et al. 2012). The identification of a wide spectrum of *NIPBL* mutations has made the molecular analysis of the *NIPBL* gene a routine component of the clinical and laboratory evaluation of patients with a suspected CdLS phenotype. Recent studies have shown that intragenic deletions in *NIPBL* are present in ∼2–5% of patient with CdLS (Bhuiyan et al. 2007; Pehlivan et al. 2012; Russo et al. 2012). Thus, the presence of *NIPBL* copy number changes has become an important factor to consider in CdLS molecular testing in suspected patients with negative *NIPBL* sequencing results. In this study we explored the occurrence of *NIPBL* copy number alterations in a cohort of 510 *NIPBL* sequence-negative patients referred to our laboratory for CdLS molecular diagnostic testing. We identified 13 cases with copy number alterations in the *NIPBL* gene, including one intragenic duplication and a deletion in the mosaic state. The size of this patient group is the largest among similar previously reported studies.

## Material and Methods

### Patient samples

The patient group consisted of 510 patients with clinical features consistent with CdLS in whom no *NIPBL* mutation was identified by sequence analysis in our laboratory. Genomic DNA was isolated from blood leukocytes on the AutoGenFlex STAR robotic workstation (Autogen, Holliston, MA) or using the MagNA Pure Compact DNA isolation system (Roche Applied Science, Indianapolis, IN) according to the manufacturer's instructions.

### Array-CGH

Deletion/duplication analysis of the *NIPBL* gene was performed using a high-resolution, exon-targeted 8X60K array-comparative genomic hybridization (CGH) platform (Agilent Technologies, Santa Clara, CA) designed to detect copy number changes in 53 genes including *NIPBL*. The array contained 2416 probes spanning the *NIPBL* gene and flanking 1-kb upstream and downstream regions with an average resolution of ∼1 probe/80 bp across the entire *NIPBL* locus. Genomic DNA samples of the patients and gender-matched controls were processed and cohybridized onto microarray slides according to the manufacturer's recommended procedures (Agilent Technologies). Microarray images were scanned at 2 *μ*m resolution and the data were extracted using ImaGene (9.0) and analyzed using the Nexus software (6.0) (BioDiscovery, Hawthorne, CA). The genomic copy number was defined by analysis of the normalized log 2 (Cy5/Cy3) ratio average of the CGH signal. Regions that reached a log 2 threshold of at least −0.32 were considered losses consistent with deletion, and thresholds of at least 0.26 were considered gains consistent with duplication.

### MLPA

Multiplex ligation-dependent probe amplification (MLPA) analysis was performed using the SALSA P141/P142 MLPA kit (MRC-Holland, Amsterdam, the Netherlands) in accordance with the manufacturer's instructions. Ligation products were polymerase chain reaction (PCR) amplified and resolved on an ABI-3730 genetic analyzer (Life Technologies, Carlsbad, CA). For quantitative analysis, peak heights of the patient and normal control were analyzed using GeneMarker Software (Soft Genetics Inc., State College, PA). Peak heights outside the range 0.7–1.3 times the control peak height were considered abnormal, with those below 0.7 representing deletions and those above 1.3 representing duplications.

### Whole-genome microarray

Whole-genome array analysis was performed using Affymetrix CytoScan HD arrays (Affymetrix, Santa Clara, CA). The CytoScan HD array contains around 2.6 million probes including 7,50,000 single nucleotide polymorphisms (SNPs) and 1.9 million nonpolymorphic markers. Whole-genome array analysis was performed according to the manufacturer's recommended protocol. Images were acquired using the GeneChip Scanner 3000 7G and analyzed using Chromosome Analysis Suite (ChAS) 1.2.3 software (Affymetrix). Human genome build 19 was used for annotation.

### Breakpoint junction sequence analyses

Break-point analysis of Patients 7 and 8 was performed by PCR primer walking using Taq polymerase and the Expand Long Template PCR System (Roche Applied Science). PCR primers were designed from the reference sequence, GenBank accession number NM_133433, across the deleted and duplicated regions derived from the array-CGH and MLPA results assuming the most likely rearrangements. PCR products were sequenced in both forward and reverse directions on an ABI 3730 DNA Analyzer (Life Technologies). Sequences were compared with the *NIPBL* reference sequence (NM_133433) using Mutation Surveyor software version 3.01 (Soft Genetics Inc.).

## Results

In total, 13/510 patients (2.5%) were found to harbor *NIPBL* structural variations (Table 1).

**Table 1:** *NIPBL* copy number variations identified by targeted CGH/whole-genome arrays.

Patient	Gender	Genotype (NCBI build 37)	Included region	CNV type	Minimum size (kb)
1	Female	chr5:g.(37005025_37005435)_(37007490_37007565)del	Ex 17–18	Deletion	2.0
2	Male	chr5:g.(36952230_36952266)_(36956007_36956380)del	Ex 2–3	Deletion	3.7
3	Male	chr5:g.(36993420_36993499)_(36997655_36997816)del	Ex 11	Deletion	4.1
4	Female	chr5:g.(37061350_37062360)_(37066620_?)del	Ex 46–47	Deletion	4.3
5	Male	chr5:g.(37061350_37062360)_(37066620_?)del	Ex 46–47	Deletion	4.3
		arr 5p13.2(37,065,152-37,089,599)x1**			24.4**
6	Female	chr5:g.(37044305_37044340)_(37049000_37049158)del	Ex 35–39	Deletion	4.7
7	Female	chr5:g.(36995835_36995861)_(37004025_37004935)del*	Ex 12–14*	Deletion	5.0
8	Female	chr5:g.(37015062_37015875)_(37021035_37021455)dup	Ex 23–27	Duplication	5.2
9	Female	chr5:g.(36968915_36969370)_(37004450_37005025)del	Ex 7–16	Deletion	35.1
10	Female	chr5:g.(36964200_36965948)_(37010235_37010791)del	Ex 7–21	Deletion	44.3
11	Male	chr5:g.(36997300_36997336)_(37066620_?)del	Ex 12–47	Deletion	69.3
		arr 5p13.2(36,994,939-37,188,352)x1**			193.4**
12	Male	chr5:g.(36900635_36903625)_(36998050_36998150)del	Ex 2–11	Deletion	94.4
13	Male	chr5:g.(?_36877675)_(37066620_?)del	Whole gene	Deletion	189.0
		arr 5p13.1p13.2(35,232,614-40,365,530)x1**			5.1 (Mb)**

* Deletion in mosaic state.

** Nomenclature/size based on SNP array results. *NIPBL* RefSeq NM_133433.

### *NIPBL* deletions

**Figure 1:**
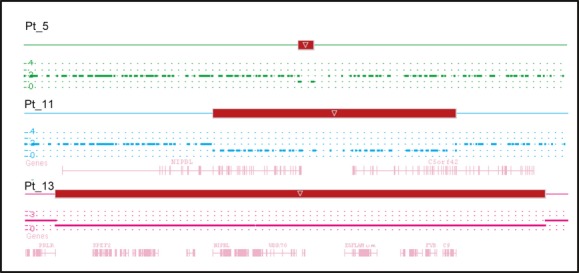
Characterization of deletions involving *NIPBL* by whole-genome microarray analysis in Patients 5, 11, and 13. In Patient 5 (Pt_5), a 24.4 kb deletion extending from the distal end of *NIPBL* into the intergenic region was observed. In Patient 11 (Pt_11), a 193.4 kb deletion was detected and extended to *C5orf42*. In Patient 13 (Pt_13), the deletion spanned 5.1 Mb and included *NIPBL* and 22 other genes. The copy number state segments (red for deletion) and copy number state data are shown for each patient. Genes involved are indicated. Not drawn to scale.

Patients 1–3 and 6 had relatively small single-and multiexon intragenic deletions ranging from ∼2 to ∼5 kb in size. Patients 9, 10, and 12 were found to have larger multiexon intragenic *NIPBL* deletions ranging from ∼35 to ∼94 kb in size. Patients 4, 5, and 11 had deletions involving the last coding exon of *NIPBL* and extending downstream of the gene. To further characterize the extent of the deletions in Patients 5 and 11, whole-genome microarray analysis was performed (Fig. 1). In Patient 5, a 24.4 kb deletion was identified at cytogenetic band position 5p13.2 that included the terminal region of *NIPBL* and extended past the gene into the intergenic region (arr 5p13.2(37,065,152-37,089,599)x1). A 193.4 kb deletion (arr 5p13.2(36,994,939-37,188,352)x1) was observed in Patient 11 and included exons 12–47 of *NIBPL* to exons 22–52 of *C5orf42* (NM_023073.3), transcribed in reverse orientation to *NIPBL*. Patient 13 had a deletion encompassing the entire *NIPBL* gene. Whole-genome microarray analysis revealed that the deletion spanned ∼5.1 Mb and involved *NIPBL* and 22 other genes at the cytogenetic band position 5p13.1p13.2 (arr 5p13.1p13.2 (35,232,614-40,365,530)x1). No additional DNA was available to further characterize the extent of the deletion detected in Patient 4.

**Figure 2:**
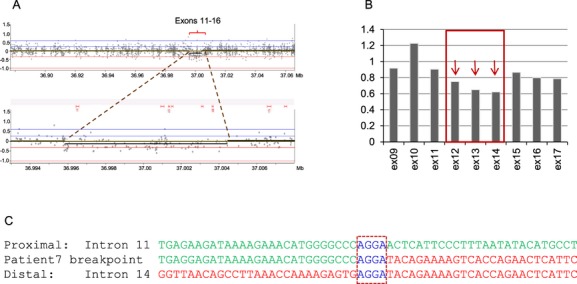
Break-point analysis of the mosaic deletion of *NIPBL* exons 12–14 in Patient 7. (A) Array-CGH profile (*top*) with the magnified region of interest (*bottom*) showing low-level reduction in the Cy5/Cy3 fluorescence log 2 ratio of oligonucleotide probes spanning exons 11–16; (B) MLPA histogram: *NIPBL* exons arrowed in red (12–14) showing reduced height ratio in comparison with control probes (∼0.7 vs. 1); (C) nucleotide sequence of the break points. Proximal reference sequence and patient break-point sequence that match with the proximal reference sequence are shown in green, whereas the distal reference sequence and patient break-point sequence that match with the distal reference sequence are shown in red. Dash boxed sequence corresponds to a region of microhomology and reveals the break-point junction.

In addition, a deletion of exons 12–14 in an apparent mosaic state was found in Patient 7. The array-CGH data showed low-level reduction in the Cy5/Cy3 fluorescence log 2 ratio of oligonucleotide probes interrogating exons 11–16 (Fig. 2A). The decreased log 2 (Cy5/Cy3) fluorescence ratios did not reach the lower defined threshold value of −0.32 and therefore this aberration was not called by the analysis software. MLPA analysis showed a ∼30% decrease in signal intensity for probes specific for *NIPBL* exons 12, 13, and 14, supporting the finding of mosaicism (Fig. 2B). Various combinations of PCR primer pairs were designed to amplify the putative deletion breakpoint junction based on the array-CGH and MLPA results. A unique 5 kb PCR product was obtained in the patient and not in the control when using primers 5′ and 3′ of introns 11 and 14, respectively. Sequence analysis of the PCR product revealed a 4968 base-pair deletion of chr5: 36,997,269-37,002,237 that included *NIPBL* exons 12–14 with a 4-bp microhomology (AGGA) at the breakpoint junction (Fig. 2C). No other tissue was available for the study.

### *NIPBL* duplications

**Figure 3:**
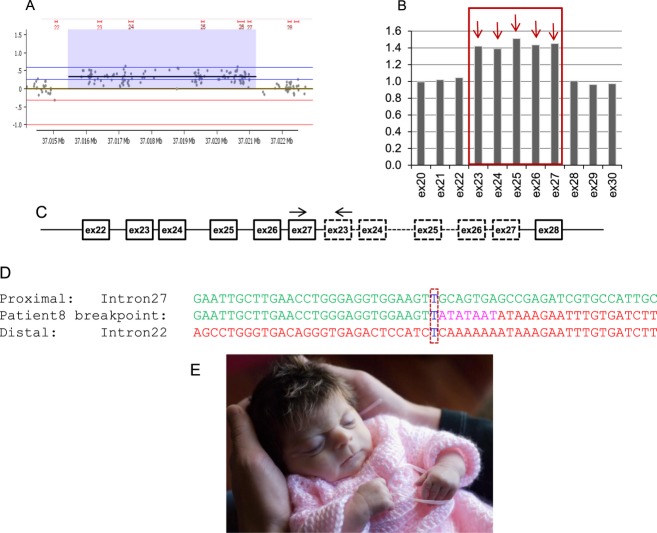
Breakpoint analysis of the duplication of *NIPBL* exons 23–27 in Patient 8. (A) Array-CGH results revealed a duplication of *NIPBL* exons 23–27; (B) MLPA histogram: *NIPBL* exons arrowed in red (23–27) showing increased height ratio (∼1.4 vs. 1); (C) schematic diagram of the duplicated region with dashed square boxes representing the exons duplicated in tandem. Arrow heads indicate the location of PCR primers used to amplify the breakpoint junction of the duplication; (D) Nucleotide sequence of the breakpoint revealing insertion (purple) of seven nucleotides (ATATAAT) and 1bp-microhomology (dash box) at the breakpoint junction; (E) Photo of Patient 8 taken at two weeks of age.

One novel multiexon *NIPBL* duplication was also identified in this cohort: a ∼5 kb duplication of *NIPBL* exons 23–27 in Patient 8. The breakpoint junction of the duplication detected by array-CGH and MLPA (Fig. 3A and B) was successfully amplified by long-range PCR using primers positioned at the very end of the duplication breakpoints, as determined by array-CGH, under the assumption that the repeated copies were arranged in tandem (Fig. 3C). Sequence analysis demonstrated that exons 23–27 were duplicated in tandem and revealed a 7-bp insertion (ATATAAT) and a 1-bp microhomology (T) at the breakpoint junction (Fig. 3D). Follow-up MLPA analysis of the patient's parents confirmed that the duplication was de novo in the patient.

### Phenotypes of CdLS patients with genomic rearrangements in *NIPBL*

Clinical information was collected for 10 of the 13 patients with *NIPBL* deletions/duplications and is presented in Table S1. Information on growth, development, craniofacial, limb abnormalities, as well as other systemic/organ involvement was requested. Age of patients ranged from 1 day to 19 years old. Complete clinical information was not available for all parameters requested and due to the young ages (1 day to 3 months) of the majority of patients, information on intellectual deficiencies and developmental delay were not fully ascertained. All reported patients had facial features consistent with CdLS, regardless of the size or location of their deletion/duplication. All patients on whom information was available had a thin upper lip, long smooth philtrum, upturned nares, and all but one had synophyrs and long thick eyelashes. All reported patients had characteristic limb abnormalities ranging from mild (fifth finger clinodactyly and 2–3 toe syndactyly) to severe (monodactyly and missing forearms). Other recurrent systems affected included cardiovascular defects, hearing loss, genitourinary anomalies, and gastroesophageal reflux, all of which are features seen in patients with CdLS.

## Discussion

**Figure 4:**
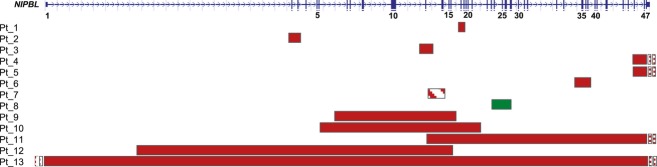
Schematic diagram of the *NIPBL* gene displaying exonic deletions and duplications in 13 patients. On top: graphic view of 47 exons (vertical blue bars) of *NIPBL*: solid horizontal bars represent *NIPBL* genomic regions deleted (red) or duplicated (green) and approximate sizes. The deletion in mosaic state in Patient 7 is indicated by a striped red bar. Narrow dotted bars indicate rearrangements extending beyond the gene in Patients 4, 5, 11, and 13. The graphical data for each patient were obtained by inputting the most distal and proximal oligonucleotide genomic probe coordinates into the custom track at the University of California, Santa Cruz website: http://genome.ucsc.edu/cgi-bin/hgGateway.

We identified 13 copy number variations (CNVs) in a cohort of 510 CdLS cases. Copy number analysis was performed utilizing high-density array-CGH targeted to the *NIPBL* gene, whole-genome SNP array, and MLPA. Deletions were found to be more common, with 12 deletions and 1 duplication identified. The CNVs ranged in size from 2 kb to 5.1 Mb with the minimum affecting one exon to the maximum affecting *NIPBL* and other adjacent genes at 5p13 (Fig. 4).

The CNVs identified in this study appear to not have been previously described in the literature supporting the broad allelic heterogeneity of *NIPBL* mutations in CdLS. An exception could be possibly represented by the 4.1 kb deletion of exon 11 observed in Patient 3 as a deletion similar in size and location has been reported previously (Pehlivan et al. 2012), and the apparently similar deletions of exons 46 and 47 identified in Patients 4 and 5. Interestingly, comparison of the sequences flanking the break points, based on array-CGH coordinates in our Patient 3 and sequence information available for the previously reported patient, revealed the presence of *Alu* sequences with ∼80% sequence similarity (*Alu*Y and *Alu*Jo) in the proximity of the borders of both deletions. Moreover, an enrichment of repetitive elements *(Alu*Jb and *Alu*Sq2) and MER2 DNA elements was also noted in the region of intron 45 (chr5: 37061350–37062360) containing the proximal breakpoint of the exons 46–47 deletions found in Patients 4 and 5. As previously suggested (Stankiewicz et al. 2003; Lupski 2007), it is possible that repetitive elements may play a role in predisposing some of these *NIPBL* regions to structural instability, although whether these motifs have any mechanistic role in the formation of some *NIPBL* deletions has yet to be determined.

Partial *NIPBL* gene deletions extending beyond the 5′ end of the gene were observed in Patients 4, 5, and 11. Whole-genome microarray analysis performed in Patients 5 and 11 (no additional DNA was available for Patient 4) revealed that the deletion in Patient 11 included the terminal part of the *C5orf42* gene. Point mutations of the *C5orf42* gene have recently been associated with autosomal recessive Joubert syndrome (Srour et al. 2012). As Joubert syndrome is a recessively inherited multisystemic disorder, we feel that its involvement in this deletion is unlikely to contribute to this patient's phenotype.

A large deletion involving the entire *NIPBL* gene was identified in Patient 13. This deletion spans ∼5 Mb in the 5p13.1-13.2 region and encompasses *NIPBL* and 22 other genes as indicated by whole-genome SNP array analysis. Physical examination of Patient 13 showed the presence of the classical craniofacial features of CdLS, bilateral upper limb reduction, genital abnormalities, bilateral hydronephrosis, Dandy Walker malformation, and developmental delay. The complexity of this patient's phenotype can be attributed to the combined haploinsufficiency of dosage-sensitive genes located within the deletion; however, the fact that the patient has classic CdLS features suggests that *NIPBL* is the major dosage-sensitive gene. Large deletions involving the *NIPBL* gene have been reported previously, and while phenotypic heterogeneity exists between patients, all display minimally diagnostic features of CdLS (Russo et al. 2012; Gervasini et al. 2013b).

Somatic mosaicism has previously been described in patients with CdLS and is not an uncommon occurrence in the mutational landscape of the *NIPBL* gene (Huisman et al. 2013). The array-CGH and MLPA data of mild-to-medium probe signal reductions infer the mechanism of somatic mosaicism for a deletion involving exons 12–14 in Patient 7. Breakpoint sequence analysis confirmed the presence of the deletion and revealed a 4-bp microhomology at the break-point junction consistent with a possible replicative mechanism such as FoSTes/microhomology-mediated break-induced replication as previously suggested (Pehlivan et al. 2012). A case of somatic mosaicism for a frameshift mutation in *NIPBL* has been reported previously in a patient showing a phenotype milder than that predicted by a truncating mutation (Castronovo et al. 2010). More recently, high level of mosaicism for a large deletion encompassing exons 2–32 of the *NIPBL* gene was identified in a patient with severe CdLS phenotype (Gervasini et al. 2013a). Our patient demonstrates typical CdLS facial features, but no severe limb reduction defects or other major abnormalities. The clinical phenotype of Patient 7 was comparable with other *NIPBL* deletion patients and was reported to be “classic CdLS” by the referring physician. A caveat is that the clinical examination of Patient 7 was done at 3 months of age; therefore, the cognitive and developmental information was limited and additional follow-up of the patient was not performed. Other tissues were not available for study and therefore we cannot exclude that the levels of somatic mosaicism might be higher in other tissues, thus leading to further functional impairments.

Intragenic duplications in the *NIPBL* gene appear to be rarer than deletions, with only one other patient harboring a single-exon duplication being reported recently (Russo et al. 2012). In this study, we identified a previously unreported de novo duplication of *NIPBL* exons 23–27 in Patient 8. Breakpoint analysis revealed the presence of a 7-bp insertion flanked by 1-bp microhomology, again suggesting a microhomology-mediated replicative mechanism as a potential predominant contributor to this rearrangement. No clinical information was available for the patient reported by Russo et al. (2012) with a duplication of exon 32 to compare with our duplication patient. 5q13 duplication syndrome [MIM#613174] is generally considered a distinct phenotype than the one observed in CdLS patients, and recently Novara et al. (2013) reported a patient with a 5p13 duplication including the *NIPBL* gene only and observed overlapping features with 5p13 microduplication syndrome. The phenotype of Patient 8 did not diverge dramatically from the classic CdLS spectrum. The patient's characteristic CdLS features included synophyrs, hirsutism, low posterior hairline, flat nasal bridge and upturned nose, thin upper lip, and downturn corners of the mouth (Fig. 3E). While 5p13 microduplication patients present with long fingers, large hands and feet, Patient 8 had small fingers and hands, and complete 2–3 toe syndactyly. In addition, Patient 8 had complete, unbalanced AV canal with hypoplastic left heart, and whereas congenital heart malformations occur in 25% of patients with CdLS, none have been reported to date in patients with 5p13 microduplication syndrome. The exons 23–27 duplication in Patient 8 is predicted to result in an out-of-frame protein and is a plausible cause of the patient's CdLS phenotype.

As CdLS is a well-described multiple malformation syndrome, we compared several CdLS facial features in our patient group with previously reported patients with *NIPBL* point mutations (Table S1). All patients for whom information was available shared the characteristic facial features of CdLS, including thin upper lip, long smooth philtrum, upturned nares, and all but one had synophyrs and long thick eyelashes. These facial features are the clinical hallmark of CdLS with synophyrs in 98%, long thick eyelashes in 99%, thin upper lip in 94%, and upturned nares in 85% of affected individuals (Kline et al. 2007). Cleft palate, which was identified in 2/7 (29%) of patients in our study, is seen in ∼20% of patients with CdLS (Kline et al. 2007). No significant differences were identified in the presence of these features in our patients with *NIPBL* CNVs and in patients with *NIPBL* point mutations (Borck et al. 2007; Schoumans et al. 2007).

Previous studies analyzing genotype–phenotype correlation in mutation-positive cases have suggested that patients with missense mutations are associated with milder phenotypes than those with truncating mutations (Gillis et al. 2004). Thus, we have further explored the possible genotype–phenotype correlation in our patient cohort. The majority of our patient cohort fell within the moderate-to-mild range (Table S2). Some correlation can be seen with regards to the size of deletion and severity of growth retardation. Patients with larger multiexonic deletions, like Patient 11 (69 kb deletion), had severe growth retardation, and patients with smaller single-exon deletions, like Patient 3 (4.1 kb deletion), had mild growth retardation. The severity pattern of limb reduction defects of our patient cohort is also similar and more in line with missense mutation patients (Table S2). One explanation of this is that several of our intragenic deletion cases (Patient 1, Patient 3, and Patient 7), which correlated with mild limb reduction defects, are predicted to result in in-frame deletions that may still lead to the formation of a NIPBL protein with some residual function. In addition, Patient 2, who also had mild limb reduction defects, has a deletion of exons 2–3. As exon 2 contains the primary start codon, it is possible that a downstream start codon at c.334 in exon 4 may possibly be utilized to initiate protein translation. While the deletion in Patient 6 is predicted to result in an out-of-frame deletion, the milder limb defects observed in this patient could be related to the smaller size of this patient's deletion and potentially to some functional aspect of the protein being preserved due to its more distal location within the gene. The large size of the deletions in Patients 9, 10, and 13 correlate with greater upper limb involvement and is consistent with the proposal that phenotypic severity is proportional to number of exons involved (Pehlivan et al. 2012). Other modifying factors, either at the *NIPBL* locus or at other genomic sites, may also play a role in the severity of limb reduction defects. Importantly, as all but three of our patients were 3 months old or younger, the lack of information regarding cognitive function is likely due to the inability to make such evaluations at the time of assessment.

In this study we further documented the heterogeneity of *NIPBL* genomic rearrangements in a cohort of 510 sequence-negative CdLS cases and identified *NIPBL* copy number aberrations in 13 (2.5%) unrelated patients. Our detection rate is lower than previously described studies, which identified *NIPBL* structural rearrangements in ∼5% of mutation-negative patients (Pehlivan et al. 2012; Russo et al. 2012). This is potentially explained by the wide phenotypic variability of patients sent to our laboratory for *NIPBL* analysis for diagnostic testing purposes. Our detection rate likely represents the true mutation detection rate of *NIPBL* copy number changes in the clinical setting.

In summary, we have shown that intragenic *NIPBL* deletion/duplication events are not uncommon in CdLS patients and result in a similar phenotype to patients with *NIPBL* point mutations. Our data contribute additional information regarding the *NIPBL* mutation spectrum in CdLS and emphasize the utility of *NIPBL* deletion and duplication analysis in the molecular diagnosis of CdLS, especially in the absence of identifiable *NIPBL* point mutations.

## Supporting Information

Table S1 Clinical features of 10 patients with copy number variations in *NIPBL.* The final column summarizes the facial features of CdLS patients identified with point mutations in *NIBPL* in comparison with the facial features of our patient cohort (second to last column).

Table S2 Limb reduction defect and growth retardation correlation of *NIPBL* copy number changes found in CdLS patients in our study.
