# Supplementary material for: Copy number analysis of NIPBL in a cohort of 510 patients reveals rare copy number variants and a mosaic deletion
Source: Mol Genet Genomic Med. 2013 Nov 14;2(2):115–23. doi: 10.1002/mgg3.48 (PMC3960053; doi:10.1002/mgg3.48)
Supplement: Table S1 — Clinical features of 10 patients with copy number variations in NIPBL. The final column summarizes the facial features of CdLS patients identified with point mutations in NIBPL in comparison with the facial features of our patient cohort (second to last column). [file mgg30002-0115-sd1.doc]

| **Patient** | **(1)** | **(2)** | **(3)** | **(6)** | **(7)** | **(8)** | **(9)** | **(10)** | **(11)** | **(13)** | **Total (%)** | **Point mutation patients (Borck et al, 2006, Shoumans et al, 2007)** |
| --- | --- | --- | --- | --- | --- | --- | --- | --- | --- | --- | --- | --- |
| **Copy number variation** | Del Ex 17-18 | Del Ex 2-3 | Del Ex 11 | Del Ex 35-39 | Del Ex 12-14 (mosaic) | Dup Ex 23-27 | Del Ex 7-16 | Del Ex 7-21 | Del Ex 12-47 | Whole gene del |  |  |
| **Age at exam and sex** | 2 days (F) | 4 mo (M) | 19 yr (M) | 10 yr (F) | 3 mo (F) | 1 day (F) | 19 mo (F) | 1 day (F) | 15 days (M) | 10 mo(M) |  |  |
| **Growth (at exam)** |  |  |  |  |  |  |  |  |  |  |  |  |
| length cm (percentile based on CdLS growth chart) |  | 55.4 (50-95th) | 161.8 (50-95^th^) | 102 (25-50^th^) | 50 (50^th^) | 45.3 (50^th^) | 64 (25-50^th^) |  | 41 (5^th^) | 62 (50-95^th^) |  |  |
| weight kg (percentile based on CdLS growth chart) |  | 4.6 (95^h^) | 53.2 (50-95^th^) | 14.4 (50-95^th^) | 3.4 (50-95^th^) | 2.1 (50^th^) | 7.8 (5-25^th^) |  |  | 6.1 (95th) |  |  |
| OFC cm (percentile based on CdLS growth chart) |  | 35.5 (5-50th) | 53.5 (98^th^) | 45 (98^th^) | 34 (25-50^th^) | 29 (2^nd^) | 42 (50-95^th^) |  | 29 (2^nd^) | 36 (25^th^) |  |  |
| **Developmental Delay** |  | + |  | + |  |  | + |  |  | + |  |  |
| **Craniofacial** |  |  |  |  |  |  |  |  |  |  |  |  |
| Synophrys | + | Mild | + | + | + | + | - | + | + | + | 9/10 (90) | 12/12 (100) |
| Long, thick eyelashes | + |  | + | + | - |  | + | + | + | + | 7/8 (88) | 6/7 (86) |
| Upturned/anteverted nares | + | + | + |  | + | + | + | + | + | + | 9/9 (100) | 5/5 (100) |
| Long, smooth philtrum | + | + | + | + | + |  | + | + | + | + | 9/9 (100) | 12/12 (100) |
| Thin upper lip |  | + | + | + | + | + | + | + | + |  | 8/8 (100) | 12/12 (100) |
| Cleft palate |  | - | - |  | - | - | - | + |  | + | 2/7 (29) | 1/5 (20) |
| **Dermatologic** |  | Hirsutism | Mild cutis marmorata | Cutis marmorata; hypoplastic nipples |  | Hirsutism | Hirsutism; hypoplastic nipples | Hirsutism; hypoplastic nipples | Hirsutism | Hirsutism |  |  |
| **Limb Abnormalities** | Proximally placed thumbs;  Disorganized toes | Small hands, 5^th^ finger clinodactyly, limitation of elbow extension;  small feet | 5^th^ finger clinodactyly,;  2-3 toe syndactyly | Small hands, limitations of elbow extension;  small feet, second toe overriding third toe bilaterally | Small hands bilaterally, small digits bilaterally | Small hands;  2-3 toe syndactyly | Underdeveloped forearm, Limitation of elbow extension;  Fifth toes overlap fourth toes bilaterally | R – monodactyly, no forearm  L-no forearm, no fingers, Limitation of elbow extension |  | Shortened arms bilateral, missing 4^th^ digit bilateral, syndactyly of 2 & 3^rd^ digit on right hand |  |  |
| **Cardiovascular** | PDA | TOF | Heart murmur | - | - | Complete, unbalanced AV canal with hypoplastic left heart, PDA, mild tricuspid regurgitation and atrial shunt | - | Coarctation of aorta, VSD |  |  |  |  |
| **Other Medical Complications** | Orbital hypoplasia w/relative proptosis of eyes; Hypoplastic genitalia; High pitched cry | Possible SNHL; Lacrimal duct obstruction; Narrow phallus | SNHL; Mild ptosis; Behavioral issues; OCD/anxiety; GER | Myopia; Spina bifida occulta; Ear infections; GER | Conductive hearing loss |  | Lacrimal duct obstruction; Myopia; Dandy Walker malformation, GER |  |  | Micropenis; Undescended testes; Grade 2 bilateral hydro-nephrosis; Dandy walker malformation, frequent pneumonia. |  |  |

**Supplementary Table I:** Clinical features of 10 patients with copy number variations in *NIPBL.* The final column summarizes the facial features of CdLS patients identified with point mutations in *NIBPL* in comparison with the facial features of our patient cohort (second to last column).

No notation indicates that the information is unavailable.

GER = gastroesophageal reflux, CDH = congenital diaphragmatic hernia, SNHL = sensorineural hearing loss, TOF = tetralogy of fallot, VSD = ventricular septal defect, PDA = patent ductus arteriosus. HLHS = hypoplastic left heart syndrome. OCD = obsessive compulsive disorder
