# Supplementary material for: Copy number analysis of NIPBL in a cohort of 510 patients reveals rare copy number variants and a mosaic deletion
Source: Mol Genet Genomic Med. 2013 Nov 14;2(2):115–23. doi: 10.1002/mgg3.48 (PMC3960053; doi:10.1002/mgg3.48)
Supplement: Table S2 — Limb reduction defect and growth retardation correlation of NIPBL copy number changes found in CdLS patients in our study. [file mgg30002-0115-sd2.doc]

**Supplementary Table 2**: Limb reduction defect and growth retardation correlation of *NIPBL* copy number changes found in CdLS patients in our study

| Limb reduction defect classification | Our patient cohort | Missense mutation patients (Gillis *et al*, 2004) | Truncating mutation patients (Gillis *et al*, 2004) |
| --- | --- | --- | --- |
| I (Mild) | 8 (80%) | 12 (92%) | 25 (60%) |
| II (Moderate) | 1 (10%) | 1 (8%) | 3 (7%) |
| III (Severe) | 1 (10%) | 0 | 14 (33%) |
| Growth Retardation |  |  |  |
| I (Mild) | 1 (11%) | 3 (33%) | 2 (7%) |
| II (Moderate) | 7 (78%) | 4 (44%) | 11 (38%) |
| III (Severe) | 1 (11%) | 2 (22%) | 16 (55%) |
